# Integration of a priori gene set information into genome-wide association studies

**DOI:** 10.1186/1753-6561-3-S7-S95

**Published:** 2009-12-15

**Authors:** Melanie Sohns, Albert Rosenberger, Heike Bickeböller

**Affiliations:** 1 Department of Genetic Epidemiology, University Medical Center Göttingen, Humboldtallee 32, 37073 Göttingen, Germany

## Abstract

In genome-wide association studies (GWAS) genetic markers are often ranked to select genes for further pursuit. Especially for moderately associated and interrelated genes, information on genes and pathways may improve the selection. We applied and combined two main approaches for data integration to a GWAS for rheumatoid arthritis, gene set enrichment analysis (GSEA) and hierarchical Bayes prioritization (HBP). Many associated genes are located in the HLA region on 6p21. However, the ranking lists of genes and gene sets differ considerably depending on the chosen approach: HBP changes the ranking only slightly and primarily contains HLA genes in the top 100 gene lists. GSEA includes also many non-HLA genes.

## Background

With genotyping chips containing 500,000 and more single-nucleotide polymorphisms (SNPs) and good genome coverage, genome-wide association studies (GWAS) are now widely used to search for susceptibility genes for complex diseases. For 500,000 statistical tests in parallel and a nominal level of 0.05, the genome-wide significance level is 10^-7^. Hence, moderately associated SNPs will have a poor chance of being found at this level, even in very large samples. Often SNPs are ranked as a first step to select a "most promising" subset of SNPs or genes to follow-up. Thus, it is of interest not to overlook so-called "gene sets" with related genes, e.g., by pathway, function, or structure, which jointly account for genomic association to the investigated trait.

Considering *p*-values for marker selection without external information may yield many false positives. Here we focus on two approaches incorporating molecular genetic knowledge, the hierarchical Bayes prioritization (HBP) by Lewinger et al. [1] and the gene set enrichment analysis (GSEA) to GWAS by Wang et al. [2]. We compare the two methods and present ways to combine them in a GWAS for rheumatoid arthritis.

## Methods

### Subjects

We applied the strategies to the genome-wide Genetic Analysis Workshop 16 (GAW16) Rheumatoid Arthritis (RA) data from the North American Rheumatoid Arthritis Consortium (NARAC). These data include 868 cases and 1194 controls, recruited to Institutional Review Board-approved protocols and genotyped on Illumina 550 k SNP chips. All research was carried out in accordance with the Declaration of Helsinki.

### GSEA and HBP

Gene set methods in GWAS are based on an initial ranking of single SNPs by *p*-values (here: Cochrane-Armitage trend test). Then they either identify biologically relevant "pathways" with functional genetic variation or they support prioritization of associated candidate markers or genes. They can be seen as enhancement to reveal the full spectrum of genes influencing disease [3].

GSEA was originally developed for gene expression analysis [4] and recently proposed for GWAS [2]. To each gene we assigned the maximal test statistic among all of its SNPs and ranked the genes from largest to smallest maximum. The enrichment score (ES) for each gene set measures if its genes are randomly distributed in the ranking or concentrated on the top. Statistical significance was assessed by permutations and family-wise error rate (FWER). The leading edge subset (LES) was defined as high-scoring genes of the significant gene sets driving the ES.

HBP [1] aims to re-rank markers using prior covariates on each marker. Regression coefficients for the relationship between prior covariates and observed single marker association statistics are estimated i) in a logistic model for the prior probability using marker distance to genes and gene set information and ii) a linear model for the strength of association. With the a posteriori probability of a marker to be associated, a re-ranked marker list is created. For GSEA we used the GenGen-package by Wang [5], for HBP we used a routine for the statistical package R provided by Lewinger et al. [1].

### Gene set and SNP annotation

For gene-to-pathway annotation (GtP), we used a file in the GenGen-package [5], and for SNP-to-gene annotation (StG), we used files from Illumina. Gene Name Service (GNS) [6] was used to assure gene name consistency. We used information from the GtP as "gene set info" and information about the physical and functional position of the SNP relative to the nearest known/predicted gene (e.g., synonymous, coding, 3'UTR) from StG as "SNP info". We combined gene sets with a large overlap and excluded sets with less than 11 genes. Finally, 876 gene sets remained.

### Strategies of data analysis with GSEA or HBP

#### I) GSEA alone

GSEA was performed on basic single-SNP association test statistics. The results are *p*-values for gene sets and a list of LES genes.

#### II) One-step HBP

HBP was performed using SNP information and gene set indicators (1 = gene in set, 0 otherwise) as prior covariates. The result is a ranking of SNPs.

#### III) Two-step HBP

HBP was performed using SNP information as prior covariate, followed by HBP additionally using gene set information. The average a posteriori probability of association of all remaining genes of the considered set was used as gene set information for all SNPs of a gene. The gene-specific probability is the maximum of the a posteriori probabilities of gene SNPs. The result is a ranking of SNPs.

#### IV) HBP followed by GSEA

HBP was performed using SNP information as prior covariate followed by GSEA using the a posteriori probabilities of HBP as entry ranking. Results are *p*-values for gene sets and a list of LES genes.

### Comparing results

GSEA assigns *p*-values to gene sets, HBP provides SNP rankings. For comparisons we restricted lists of genes to 100 and lists of gene sets to 20. To compare the **most promising genes**, we considered all LES genes of best-ranked GSEA sets. For Strategies II and III, the highest ranked SNP per gene was used for a ranked top-gene list. For Strategies I and IV, we compared the **ranking of identified gene sets **according to their *p*-value; for Strategies II and III, according to the corresponding gene set regression parameters of the logistic submodel. To quantify the overlap of identified genes/gene sets, we calculated an overlap-index as ratio between observed different list elements relative to the total number of list positions l_list _.

This index is 0 if all elements are different, and 1 if all lists contain the same elements. It may be roughly interpreted as the chance of an element to appear in another list as well.

## Results

After quality control and trend test, 334 SNPs are significant at the genome-wide level. They belong to 90 genes (81 in HLA region) that are involved in 153 gene sets. Due to computer limitations we had to restrict the number of considered pathways to 100, thus using only the top 75 genes. This led to only two a priori gene sets without genes from the HLA-region, and hence to an influence on the preference towards HLA.

Strategy I (GSEA) yielded 20 gene sets with FWER < 0.05. The 19 best-ranked gene sets contained the top 100 LES genes. Strategy IV (HBP+GSEA) resulted in three gene sets with FWER < 0.05. The two best-ranked gene sets contained 68 LES genes. Only subset LES genes of the third gene set could be added to fill up to 100 top genes.

### Comparison of most promising genes

**Figure 1:**
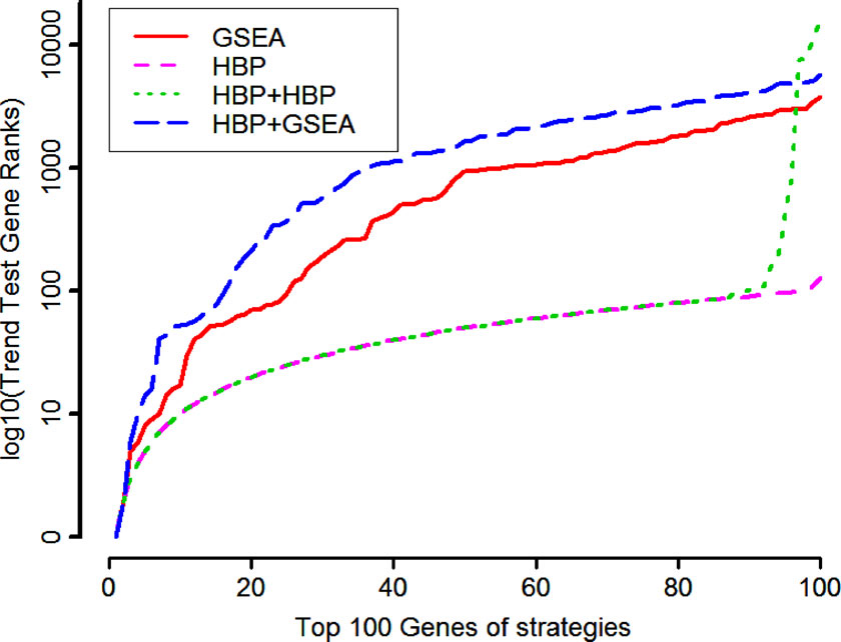
**Gene ranks after applying GSEA, one/two-step HPB or HBP+GSEA**. The top 100 genes were ordered by initial trend test *p*-values.

A comparison of all four strategies by the ranks of the top 100 genes with those of the initial ranks is given in Figure 1. The general overlap-index for the gene lists (I_g_) is I_g_(I, II, III, IV) = 0.51. The lists we obtain by either of the two HBP-only strategies are almost identical (I_g_(II, III) = 0.89) and are almost unchanged compared with the initial list (I_g_(II, III, init) = 0.94). In Strategy II only two new genes appeared; in Strategy III, 11. However, the LES of the two GSEA strategies highlighted many new genes. Only 25 or 16 of the initial top 100 genes (primarily HLA), respectively, are included in the top 100 genes of Strategy I and IV, leading to a low overlap with the initial list (I_g_(I, IV, init) = 0.31). The new LES genes initially had ranks of up to 21,361. However, the LES gene lists of these two strategies also differ remarkably (I_g_(I, IV) = 0.37), with only 37 genes in common. The initial ranking reveals the extraordinary importance of the HLA region. Because HBP changed this only slightly, both HBP strategies contain more than 80 HLA genes in their top 100 gene lists. Apart from 29 or 19 HLA genes, respectively, the GSEA strategies include many non-HLA genes, which could be a new starting point to identify yet unknown additional genes influencing the disease.

### Comparison of gene set ranking

In total, the four top 20 lists comprise 51 different gene sets. The general overlap-index for the gene sets (I_s_) is I_s_(I, II, III, IV) = 0.48. Only two gene sets appear in all four lists: "GO0002460: adaptive immune response" (66 genes) and "hsa04612: antigen processing and presentation" (61 genes). "hsa04612" contains 22 genes of the HLA region, while "GO0002560" contains only 3. The non-HLA genes in this latter set are responsible for activation or inhibition of immune reactions so that this identified set is a reasonable candidate for RA. These two gene sets share only three genes. Interestingly, hsa04612 reached high ranks (third and fourth) only at those strategies with a GSEA step, but ranks 11 and 17 when HBP was the final step. On the other hand, GO0002460 ranked between the 11^th ^and the 19^th ^rank in all four strategies. Comparing the top 20 gene sets (Table 1) we see that Strategy II yields essentially different results, with list-to-list overlap-indices of I_s _= 0.25. The remaining strategies comprise 38 gene sets with overlap-index of I_s_(I, III, IV) = 0.55. They have 7 gene sets in common, that are composed of between 11 and 63 genes, but share pairwise no more than 5 genes. Directly comparing Strategies I and IV and Strategies II and III, respectively, results in overlap-indices of I_s_(I, IV) = 0.55 and I_s_(II, III) = 0.15. This indicates a more robust ranking of gene sets by GSEA than by HBP. But even within the top 20 gene sets of I and IV, the ratio between shared and non-shared gene sets is 11:18. Changing the number of top gene sets to consider leads to similar results even though the exact number of sets identified in all four lists changes (data not shown). Although the top 100 gene lists of both strategies with HBP as last step are almost identical, the top gene sets diverge considerably.

**Table 1:** Top gene sets after applying GSEA, one/two-step HPB, or HBP+GSEA

rank	Strategy I: GSEA	Strategy II: HBP	Strategy III: HBP+HBP	Strategy IV: HBP+GSEA
1	hsa04514 ^a^	hsa04330 ^a^	GO0032393 ^a^	hsa04940 ^b^
2	hsa04640	GO0032395^b^	GO0002504 ^b^	hsa04514 ^a^
3	hsa04612 ^c^	GO0006956 ^a^	GO0048002 ^b^	GO0008236
4	hsa04940 ^b^	GO0016820	GO0051327	hsa04612 ^c^
5	inflamPathway	GO0051028	asbcellPathway ^b^	GO0032395^b^
6	th1th2Pathway ^a^	GO0004004	GO0042287	GO0002504 ^b^
7	CSKPathway	GO0030554	GO0032395^b^	GO0048002 ^b^
8	ctla4Pathway^b^	GO0000279	GO0001569	GO0051249 ^a^
9	blymphoPathway	GO0051276	GO0051249	hsa04512 ^a^
10	hsa04650 ^a^	GO0019199	GO0002526 ^b^	GO0032393 ^a^
11	tcraPathway	GO0002460 ^c^	GO0006957	th1th2Pathway ^a^
12	GO0048002 ^b^	GO0007160	GO0002460 ^c^	hsa04650 ^a^
13	GO0046982	hsa04940 ^b^	ctla4Pathway ^b^	hsa04610
14	GO0009405	hsa04612 ^c^	hsa00310	GO0002526 ^b^
15	asbcellPathway ^b^	hsa04010	hsa04512^a^	GO0002460 ^c^
16	hsa04330 ^a^	GO0043069	GO00051169	ctla4Path ^b^
17	GO0006956 ^a^	GO0002521	hsa04612 ^c^	GO0002443 ^a^
18	GO0002504 ^b^	GO0002443 ^a^	hsa04320	GO0004175
19	GO0002460 ^c^	GO0006281	GO0006643	asbcellPathway ^b^
20	GO0002526 ^b^	GO0009952	GO0016301	GO0003779

^a^in two strategies

^b^in three strategies

^c^in all four strategies

## Discussion

GWAS aim to discover new associations and novel disease genes. For complex diseases, many potentially interacting genes may be involved. Biological processes, indicated by gene sets rather than single genes, might warrant further investigation.

The gene set approaches cannot replace the original GWAS ranking, but they may identify additional SNPs within sets that escaped identification due to weak marginal effects. Locus heterogeneity within one pathway, also a possible replication problem, can be considered. Gene set approaches can help to structure results and to distinguish truly associated from unassociated markers [3]. In this context please note that not all biological details can be incorporated, especially because many gene sets are not yet well understood and updates in databases lag behind knowledge.

In this GWAS, 87 out of the top 100 initial ranked genes are in the HLA region, a region well known for its role in RA. The special challenges are to contrast genes within HLA region, but also to identify non-HLA susceptibility genes.

Neither GSEA nor HBP is a gold standard for the integration of gene set information into GWAS. We found considerable differences in the resulting lists of the most promising genes and gene sets. The chance of a gene appearing in more than one of our final gene lists is only 50%. The same is true for gene sets. Although the top 100 gene lists of both approaches with only HBP are almost identical, their lists of gene sets overlapped in only 3 of the top 20 entries. These heterogenous results point to methodological differences. GSEA uses the ranking of genes to find enriched gene sets by summing ranks, while HBP uses prior gene set information to change the ranking of SNPs. Hence, GSEA directly leads to list of most promising gene sets and only builds a bridge by LES to a list of genes. For HBP, the reverse is true.

In GSEA, genes with many SNPs are favored by using the maximal test statistic per gene. In HBP, considering all markers of a gene may penalize larger genes, because a true association signal at one marker might be diluted by all unassociated markers of the gene. GSEA corrects for linkage disequilibrium structure and for multiple testing by false-discovery rate or FWER by a computationally intensive permutation procedure. Neither correction is considered for the much faster HBP.

Strategies II and III differ only in the way gene set information is prepared for HBP. In II we used an indicator for a gene set as "prior" information, for III we used set-specific weights derived from the observed association, which is not strictly "prior".

In comparison with single-SNP analysis, HBP can "be superior when the proportion of true positive associations is not too small, as in GWAS with hundreds of truly associated SNPs" [1]. This can explain the difference in identified gene sets when compared with GSEA. Lewinger also stated that "when the non-centrality parameters of the true associations are large enough to be picked by the raw test statistics there is little to be gained from prior covariates" [1]. Hence, in this GWAS with 334 genome-wide significant SNPs, the list of most promising genes did not change for HBP. However, with GSEA new non-HLA genes were identified. The methods have a substantial influence on the re-ranked list of top genes. Strategies I and IV incorporate significance of gene sets, while Strategies II and III use regression coefficients for selecting the top sets, which provide only information on the magnitude of up-ranking of the genes included in the gene sets.

Note that combining different ranking lists - not considered here - may lead to a so-called voting paradoxes.

In summary, HBP keeps the prominent role of the HLA-complex while GSEA enriches the top gene list with non-HLA genes. Both methods identified the well known association of HLA and RA. The finding of non-HLA SNPs by the GSEA suggests that HLA and non-HLA markers are involved in the disease process. All strategies included the sets GO0002460 and hsa04612 in their top 20 gene set lists. Thus, all have the ability to recognize HLA-dominated gene sets as well as other sets. Because both approaches have their own rationale, the choice of the method is currently a matter of preference. The main advantage of HBP over GSEA is that different types of prior information can be considered, not only gene set information.

The considered methods were developed to increase signals jointly for weakly informative markers in different genes but within one gene set. Because GSEA uses only the maximal SNP test statistic per gene, several weakly informative markers within one gene will not be detected. This problem may be addressed by combining the SNP statistics with one-gene statistics or by processing SNP sets instead of gene sets. We concentrated on single-SNP methods. Please note that depending on the context, haploype approaches or machine learning methods might be more advantageous.

## Conclusion

Considering prior information, e.g., sets of biological interrelated genes, is a promising method in GWAS analysis. Some critical aspects still need to be examined, including whether to reduce the set of markers and how. The chosen method has a large impact as the resulting lists of "most promising" genes or gene sets may be very different.

## List of abbreviations used

ES: Enrichment score; FWER: Family-wise error rate; GAW16: Genetic Analysis Workshop 16; GSEA: Gene set enrichment analysis; GNS: Gene Name Service; GtP: Gene-to-pathway annotation; GWAS: Genome-wide association study; HBP: Hierarchical Bayes prioritization; LES: Leading edge subset; NARAC: North American Rheumatoid Arthritis Consortium; RA: Rheumatoid arthritis; SNP: Single-nucleotide polymorphism; StP: SNP-to-gene annotation.

## Competing interests

The authors declare that they have no competing interests.

## Authors' contributions

AR processed the SNP-to-gene and the gene-to-pathway annotation. MS performed the statistical analysis. All authors participated in interpretation of the results and writing of the manuscript. All authors read and approved the final manuscript.
